# MAFLD Pandemic: Updates in Pharmacotherapeutic Approach Development

**DOI:** 10.3390/cimb46070376

**Published:** 2024-06-21

**Authors:** Farah Khaznadar, Omar Khaznadar, Ana Petrovic, Marija Hefer, Fabian Gjoni, Stefan Gjoni, Justinija Steiner, Martina Smolic, Kristina Bojanic

**Affiliations:** 1 Faculty of Dental Medicine and Health Osijek, Josip Juraj Strossmayer University of Osijek, 31000 Osijek, Croatia; farah.khaznadar.8@gmail.com (F.K.); anapetrovic@fdmz.hr (A.P.); mhefer@fdmz.hr (M.H.); msmolic@fdmz.hr (M.S.); 2 Faculty of Medicine Osijek, Josip Juraj Strossmayer University of Osijek, 31000 Osijek, Croatia; 3 Department of Radiology, “Dr. Juraj Njavro” National Memorial Hospital Vukovar, 32000 Vukovar, Croatia; khaznadar.omar@gmail.com; 4 Opća bolnica Pula, Santoriova ul. 24a, 52100 Pula, Croatia; fabiangjoni@yahoo.com (F.G.); stefan.gjoni@yahoo.com (S.G.); 5 Health Center Osijek-Baranja County, 31000 Osijek, Croatia; justinija.pavkov@gmail.com

**Keywords:** MAFLD, THR-β agonists, FGF-21 agonists, SGLT2 inhibitors

## Abstract

With around one billion of the world’s population affected, the era of the metabolic-associated fatty liver disease (MAFLD) pandemic has entered the global stage. MAFLD is a chronic progressive liver disease with accompanying metabolic disorders such as type 2 diabetes mellitus and obesity which can progress asymptomatically to liver cirrhosis and subsequently to hepatocellular carcinoma (HCC), and for which to date there are almost no approved pharmacologic options. Because MAFLD has a very complex etiology and it also affects extrahepatic organs, a multidisciplinary approach is required when it comes to finding an effective and safe active substance for MAFLD treatment. The optimal drug for MAFLD should diminish steatosis, fibrosis and inflammation in the liver, and the winner for MAFLD drug authorisation seems to be the one that significantly improves liver histology. Saroglitazar (Lipaglyn^®^) was approved for metabolic-dysfunction-associated steatohepatitis (MASH) in India in 2020; however, the drug is still being investigated in other countries. Although the pharmaceutical industry is still lagging behind in developing an approved pharmacologic therapy for MAFLD, research has recently intensified and many molecules which are in the final stages of clinical trials are expected to be approved in the coming few years. Already this year, the first drug (Rezdiffra™) in the United States was approved via accelerated procedure for treatment of MAFLD, i.e., of MASH in adults. This review underscores the most recent information related to the development of drugs for MAFLD treatment, focusing on the molecules that have come furthest towards approval.

## 1. Introduction

The global prevalence of metabolic-associated fatty liver disease (MAFLD), estimated to be around one billion, is rapidly increasing, hand in hand with the growing prevalence of obesity and type 2 diabetes mellitus (T2DM) [1,2,3]. MAFLD is a chronic progressive disease marked by an excessive accumulation of fat in the liver (5% or above of liver’s weight) associated with a metabolic disorder such as obesity/overweight and insulin resistance [4,5]. MAFLD was known formerly as non-alcoholic fatty liver disease (NAFLD). Based on the many study reports indicating that the majority of NAFLD patients also have some type of metabolic disorder, such as T2DM, insulin resistance, obesity, dyslipidaemia or hypertension, and conversely that the majority of patients with a metabolic disorder will sooner or later develop NAFLD, it became clear that NAFLD and metabolic disorder share a common pathological pathway and are practically inseparable conditions [1,6]. Aiming to better capture the underlying pathogenesis and affected patients and to reshape treatment strategies, it was proposed that NAFLD be renamed as MAFLD in 2020 by a group of world-leading hepatology experts [7]. The focus of this new terminology is on the inclusion of “positive” disease criteria for MAFLD diagnosis: overweight/obesity or T2DM or at least two metabolic risk factors in patients having normal/lean weight defined by the criteria for the patient’s specific ethnic group, which should be present in addition to evidence of steatosis detected via biopsy, imaging or biomarkers in the blood [8]. Moreover, the MAFLD definition does not rule out patients with excessive alcohol consumption or with another type of chronic liver disease [7,9]. After the new terminology was proposed, several studies aimed to objectively investigate the utility of this renaming [10,11].

**Figure 1:**
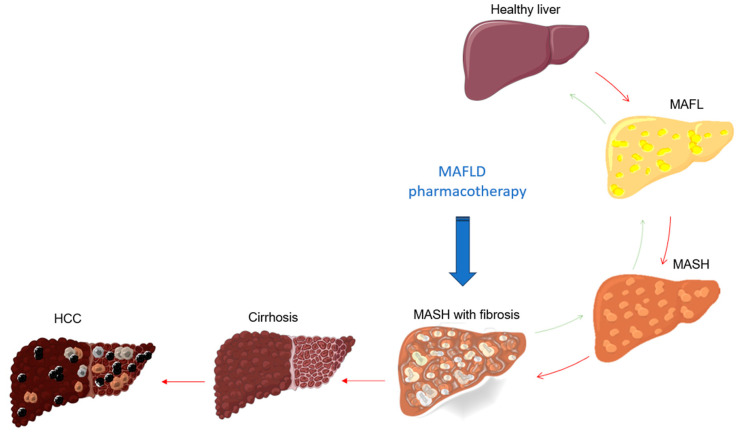
Exacerbation of MAFLD and pharmacotherapy to reverse the stage of fibrotic MASH to a healthy liver. MAFLD: metabolic-associated fatty liver disease, MAFL: metabolic-associated fatty liver, MASH: metabolic-associated steatohepatitis, HCC: hepatocellular carcinoma. Red arrow: worsening liver condition. Light green arrow: improving MAFLD stages. Blue arrow: target liver condition for MAFLD pharmacotherapy. Figure created with Servier Medical Art, https://smart.servier.com/ (accessed on 4 May 2024).

In a study by Lin et al. in 2020, MAFLD and NAFLD criteria were compared and it was found that the MAFLD terminology emerges as more practically feasible for identifying patients at higher risk of disease exacerbation [10]. Another study conducted on 3709 patients in Japan reported recently in 2024 that the prevalence of MAFLD in NAFLD patients was 96.7% and that waist circumference criteria for NAFLD and metabolic syndrome matched 96.2%, leading the authors to conclude that in the Japanese population, patients with NAFLD can be reclassified as having MAFLD [11]. Generally, MAFLD is considered to be caused by a combination of various risk factors such as metabolic syndrome, oxidative stress, gut microbiota imbalance and genetic factors such as genetic polymorphisms and epigenetic alterations [1]. MAFLD encompasses a diverse range of liver diseases and can progress from simple steatosis, i.e., metabolic-associated fatty liver (MAFL), to metabolic-associated steatohepatitis (MASH), cirrhosis and hepatocellular carcinoma (HCC), which is one of the most leading causes of liver transplantation worldwide (Figure 1) [12,13,14]. Unfortunately, MAFLD can remain undetected for years because the symptoms often occur very late, when the patient has already developed cirrhosis [15]. Due to this complex multifactorial etiology, the molecular mechanisms and biomarkers involved in MAFLD are not yet fully understood, making the search for an appropriate pharmacological treatment challenging [16].

The primary treatment approach in MAFLD patients is to integrate a healthy lifestyle including a Mediterranean diet, physical activity and weight loss, which can contribute to the reduction of liver steatosis and fibrosis [2,17]. However, studies have shown that most of the patients cannot reach the target weight required to reduce liver fibrosis, which is the most important mortality prognostic factor in MAFLD [17]. It is worth emphasizing that a normal BMI does not indicate a healthy metabolic status of the patient, as MAFLD can also occur in lean patients. These patients have a poor metabolic profile compared to the healthy population in regard to elevated blood pressure, glucose, HbA1c, triglycerides, LDL and decreased HDL [18,19]. All in all, the non-pharmacological approach is essential but not sufficiently effective as a stand-alone measure. Consequently, there in an urgent need for an approved pharmacological treatment, which is not yet available, with the exception of a just recently approved drug [2,20]. Namely, in March 2024, a thyroid hormone receptors β (THR-β) agonist, resmetirom (Rezdiffra™), became the first drug approved by the Food and Drug Administration (FDA) in the United States for the treatment of non-cirrhotic MASH in adults with moderate to progressed liver fibrosis in combination with diet and exercise [20].

The insight into the current most promising therapeutic options that could be integrated into daily clinical practice and guidelines for MALFD in the near future will be further discussed in this article.

### Pathophysiology of Liver Fibrosis

Through its anti-lipolytic function in lipid metabolism, insulin promotes deposition of free fatty acids (FFAs) and triglycerides (TGs) in adipose tissue [21,22]. Under condition of insulin resistance, insulin is unable to inhibit adipose lipolysis, which in turn leads to the release of FFAs from adipose tissue and their excessive accumulation and formation of TGs in liver [21]. For this reason, insulin resistance is one of the leading causes of fatty liver and has become one of the important therapeutic targets in MAFLD treatment [23,24]. Most of the FFAs (60%) in the liver come from adipose tissue, while the rest from hepatic de novo *lipogenesis* and from diet. Excessive accumulation of FFAs is considered to be the main trigger of the pathogenic pathway of liver fibrosis [17]. Surplus of FFAs in the liver generates lipotoxic lipids that cause endoplasmic reticulum stress, oxidative stress, inflammation and apoptosis of hepatocytes, resulting in formation of reactive oxygen species [25,26].

In such an environment, Kupffer cells (macrophages in the liver sinusoids) release the profibrotic factor transforming growth factor-β (TGF-β), which activates the hepatic stellate cells. Engaged hepatic stellate cells then migrate to the site of injury, secrete an extracellular matrix and form a fibrotic tissue in the liver [17,26,27]. While fibrinogenesis, i.e., the formation of a “scar” in the wound, is a normal physiological healing process, it becomes pathogenic if it occurs persistently [15]. Grade of liver fibrosis reflects the severity of liver damage, and extensive liver fibrosis is a main hallmark of compensated or uncompensated cirrhosis and HCC (Figure 1) [28,29]. The assessment of liver fibrosis in MAFLD patients could therefore be used for the evaluation of treatment response [30].

## 2. Latest Updates in MAFLD Treatment

### 2.1. Thyroid Hormone Receptor β (THR-β) Agonists

The thyroid gland secretes thyroid hormones, which are key regulators of numerous physiological processes such as cell growth, fetal development and carbohydrate, protein and fat metabolism, and therefore these hormones have an impact on practically every organ, in particular on the liver [31,32]. Stimulation of thyroid hormone receptors β (THR-β), which are mainly expressed in the liver, by thyroid hormones enhances lipid metabolism and FFA mobilization, leading to a reduction in low-density lipoprotein (LDL) cholesterol and TG levels, hepatic steatosis and fibrosis [26,32,33]. Clinical evidence for this close relation between thyroid hormones and MAFLD could include the fact that hypothyroidism is more common in patients with MAFLD [34,35]. Additionally, in patients who progress to MASH, in parallel with the steatosis increasing, the activity of THR-β receptors in liver decreases, i.e., the receptors become less sensitive to thyroid hormones [36].

With the aim to improve liver condition, a drug targeting the liver, the THR-β agonist resmetirom (Rezdiffra™), was developed. Compared to triiodothyronine (T3), this orally administered drug is around 28 times more selective for THR-β than for THR-α and has low uptake in extrahepatic tissues [34,37]. Based on the previously conducted open-label extension study (OLE, NCT02912260), which enrolled 31 patients with mildly elevated liver enzymes and revealed a reduction in fibrosis, LDL and TG levels in these patients, the phase 3 study called MAESTRO-NASH (NCT03900429) was initiated [26,34]. This ongoing 54-month study is designed as a placebo-controlled, double-blind RCT (randomized clinical trial) and has enrolled a total of 966 patients until week 52. The biopsy results demonstrated that compared to the placebo group, in which patients were advised on healthy nutrition and exercise, a larger number of patients on resmetirom therapy showed resolution or no aggravation of MASH or liver fibrosis [38,39]. Harrison et al. reported that 25.9% of patients on 80 mg resmetirom therapy and 29.9% of patients on 100 mg resmetirom therapy showed resolution of MASH and no aggravation of liver fibrosis versus 9.7% of patients in the placebo group. Additionally, patients in the 80 mg and 100 mg resmetirom group (24.2% and 25.9%, respectively) showed benefit in liver fibrosis and no aggravation of MASH in comparison with the placebo group (14.2%) [38,39]. Following the publication of significant results after one year of the MAESTRO-NASH study, the US FDA granted accelerated approval of resmetirom under the trade name Rezdiffra in March 2024 for the indication of non-cirrhotic MASH in adult patients with mild to advanced liver fibrosis (corresponding to fibrosis stages F2 to F3) in combination with diet and physical activity [20,39]. So far, Rezdiffra has demonstrated a good safety profile, with diarrhea and nausea reported as the most common side effects. However, the sponsor still must complete 54 months of this study in order to demonstrate clinical benefit in terms of liver-related outcomes along with an acceptable safety profile [39,40].

Another orally administered THR-β agonist targeting the liver, VK2809, is still under investigation and has not yet reached the approval phase but shows promising potential [41,42]. Its efficacy and safety have been evaluated in two double-blind, randomized clinical trials (RCTs): a 12-week phase 2a study (NCT02927184), which was completed in 2019, and an ongoing 52-week phase 2b study (VOYAGE, NCT04173065), which is expected to be completed in June 2024 [43,44]. The phase 2a study was conducted in 59 patients with MAFLD and hypercholesterolemia and showed a significant reduction in LDL-C, other hepatic lipid content (such as lipoprotein A and apolipoprotein B) and alanine aminotransferase (ALT) levels. In May 2023, at 12 weeks of the phase 2b study conducted in patients with MASH and fibrosis (biopsy-proven), the primary endpoint was met as the results showed a decreased hepatic fat content [26,42]. So far, both studies have shown a good safety profile with mostly mild adverse events (AEs) reported [42].

### 2.2. Fibroblast Growth Factor 21 (FGF-21) Agonists

The action of the hormone fibroblast growth factor 21 (FGF-21) in the liver leads to a reduction in liver fat, as it stimulates fatty acid oxidation and the secretion of triglycerides and very low-density lipoproteins (VLDL) and inhibits de novo *lipogenesis* [45]. Due to the short half-life of human FGF-21, the development of FGF-21 analogs requires structural modifications to increase stability and avoid rapid elimination from the body [46]. An FGF-21 analog, pegozafermin (BIO89-100), was developed as subcutaneous injection for the therapy of MASH as well as severe hypertriglyceridemia [47]. Being pegylated, pegozafermin has a prolonged half-life compared to FGF-21, so it only needs to be administered once every 14 days [45]. Loomba et al. (2023) reported that in a phase 2b placebo-controlled RCT (NCT04929483) which enrolled a total of 222 patients, pegozafermin demonstrated fibrosis improvement in patients with MASH [48]. Based on these encouraging results, pegozafermin entered phase 3 clinical trials (NCT06318169) in March 2024 to evaluate the safety and efficacy of pegozafermin in patients with MASH and fibrosis. A total of 1050 patients will be enrolled in the study, which is expected to be completed in 2029 [47]. Efruxifermin, which exerts an agonistic effect on FGF-21, is another promising drug from this group. This drug is a fusion protein with increased stability in the body, which consists of a human IgG1-Fc domain and two altered FGF-21 [49]. The first results of the placebo-controlled phase 2b RCT (NCT04767529) named HARMONY were published in December 2023. This study was conducted in patients with MASH with fibrosis stage F2 or F3, with the primary endpoint of assessing improvement in at least one fibrosis stage without worsening of MASH after 24 weeks. Analysis of this part of the study showed that this outcome was achieved in 19% of patients in the placebo group compared to 36% of patients in the 28 mg efruxifermin group and 33% in the 50 mg efruxifermin group, leading the study investigators to the conclusion that efruxifermin improves liver fibrosis [49,50,51]. In light of these favorable findings, efruxifermin has entered the two ongoing placebo-controlled phase 3 RCTs that began in late 2023. One of these is the SYNCHRONY histology study (NCT06215716), which is expected to recruit 1000 participants and to be finalized by March 2027. The main goal of this study is to investigate the improvement of at least one grade of liver fibrosis with MASH resolution after 52 weeks of treatment with efruxifermin. A further RCT called SYNCHRONY Real-World (NCT06161571) will investigate the safety and tolerability of efruxifermin in 700 MAFLD patients until October 2026 [52].

### 2.3. Incretin and Glucagon Receptor Agonists

The endogenous incretin hormones glucagon-like peptide-1 (GLP-1) and glucose-dependent insulinotropic polypeptide (GIP) are secreted by the L-cells and K-cells of the intestine and are responsible for very strong insulin secretion after a meal [53,54]. A viable therapeutic option for the treatment of MAFLD is the antidiabetic drug semaglutide, which is available on the market either as a subcutaneous injection or as an oral drug [55]. Semaglutide is an agonist of the GLP-1 receptor which, when binding to the GLP-1 receptor, triggers various signaling mechanisms that lead to insulin secretion and a reduction in glucagon, which in turn results in a reduction in blood glucose levels [56]. This drug is also approved for the treatment of obesity, as it can promote weight loss by regulating appetite and inhibiting gastric emptying [56,57]. Last year, a meta-analysis by Zhu et al. reported that semaglutide significantly reduced hepatic steatosis, inflammation, hepatocellular ballooning and liver stiffness, while the effect on reducing fibrosis stage is still uncertain [58]. According to RCTs (NCT02970942 and NCT03357380), semaglutide demonstrated positive effects on the liver as it decreased ALT level, liver inflammation and steatosis [59,60]. Semaglutide is currently being investigated in the ESSENCE phase 3 clinical study designed as a randomized, placebo-controlled trial (NCT04822181), which is enrolling a total of 1200 patients and is expected to be completed by 2029. The study investigates MASH outcomes without fibrosis exacerbation and improvement of liver fibrosis without MASH exacerbation in non-cirrhotic MASH patients [61].

The dual GLP-1 and glucagon receptor agonist efinopegdutide (MK-6024) is a subcutaneously administered drug that was developed for the treatment of MAFLD and is currently undergoing clinical trials [62]. This drug has already obtained fast-track designation from the US FDA. This is a procedure aimed to accelerate the development and evaluation of drugs for the treatment of serious conditions in order to meet urgent medical needs as soon as possible [63]. In a randomized phase 2a clinical trial (NCT04944992), the efficacy of efinopegdutide in reducing hepatic fat in MAFLD patients was compared with that of semaglutide, resulting in a significantly greater reduction in hepatic fat with efinopegdutide than with semaglutide [62]. In view of these promising results, the investigation of efinopegdutide continues in a randomized, double-blind, placebo-controlled phase 2b clinical trial (NCT05877547). The aim of this ongoing study, with an estimated duration until the end of 2025, is to assess its efficacy in resolution of MASH without aggravation of liver scarring in a total of 300 non-diabetic patients with histologically proven precirrhotic MASH [63,64]. Tirzepatide is a novel drug that is approved for T2DM and obesity and is being intensively trialed for other indications, including MAFLD, as it has already shown beneficial effects on MAFLD biomarkers in patients with T2DM. This drug belongs to the “twincretins” group as it is a dual GLP-1 and GIP receptor agonist [54,65,66]. At the beginning of 2024, the phase 2 RCT SYNERGY-NASH (NCT04166773) was completed, which was conducted in MASH patients to evaluate the safety and efficacy of tirzepatide. The primary endpoint was to determine whether tirzepatide leads to resolution of MASH and no worsening of fibrosis, and the secondary endpoint was the change in fibrosis stage, liver fat content and body weight [54]. The study sponsor announced the positive results of the clinical trial and reported about up to 74% of patients meeting the primary endpoint, compared to around 13% in the placebo group. However, the detailed study results are yet to be published [67].

### 2.4. Sodium-Glucose Cotransporter 2 (SGLT2) Inhibitors

**Figure 2:**
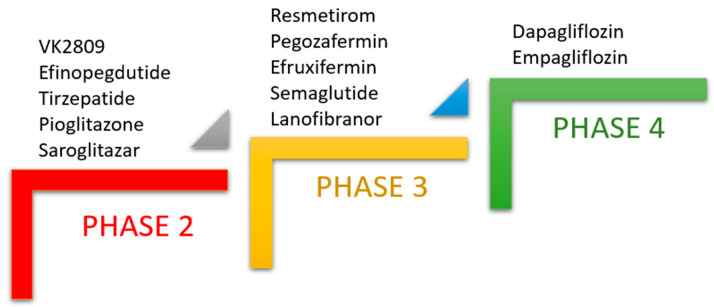
Molecules trialed for metabolic-associated fatty liver disease sorted by their most current randomized clinical trial phase.

One of the top candidates for treatment of MAFLD are oral antidiabetic drugs sodium-glucose cotransporter 2 (SGLT2) inhibitors, commonly called “flozins” [1,68,69]. They lower blood glucose by inhibiting SGLT2 in the proximal renal tubule, thus blocking the reabsorption of glucose into the bloodstream and promoting its excretion via the urine [68]. SGLT2 inhibitors are generally considered safe; the most commonly reported adverse effects are infections of the genitourinary tract, hypotension and diabetic ketoacidosis, which are associated with their mechanism of action [70]. Furthermore, SGLT2 inhibitors have recently been found to have cardio- and kidney-protective effects due to their antiphlogistic and antifibrotic mode of action, which has led to their marketing authorization for non-diabetic indications, namely heart failure and chronic kidney disease [71,72,73]. Reduction of inflammation, steatosis and fibrosis have been suggested as beneficial effects on the liver, for which the clinical efficacy of SGLT2 inhibitors is being investigated in numerous RCTs [1,74,75]. Among all SGLT2 inhibitors, dapagliflozin and empagliflozin have come the longest way (Figure 2) [65]. The safety of dapagliflozin is still being assessed in a placebo-controlled phase 3 RCT (NCT05308160), which is expected to enroll a total of 75 patients with MAFLD diagnosed with steatosis grade 2 or higher detected by FibroScan device, and to be finished by the end of April 2024 [65,76]. In another placebo-controlled phase 3 RCT, the so-called DEAN study (NCT03723252), a total of 154 patients with biopsy-confirmed MASH and metabolic risk factors were recruited in China, with the primary endpoint being histological improvement after 12 months of dapagliflozin therapy. Secondary endpoints included MASH resolution, liver fibrosis and steatosis, inflammatory biomarkers and metabolic factors (weight, waist circumference, blood pressure, HbA1c). This study has just been completed and the results of the study are still being awaited [61,77,78,79]. An ongoing phase 4 clinical trial (NCT05459701) evaluating the effect of dapagliflozin on the liver of patients with type 2 diabetes mellitus and MAFLD compared to the placebo group over a period of 6 months, with leptin, adiponectin and vascular cell adhesion molecule 1 (VCAM-1) as primary outcomes, is expected to end in an upcoming period [80,81]. A double-blind, placebo-controlled phase 4 RCT (NCT04642261), which had the primary goal of assessing whether empagliflozin can reduce liver steatosis measured by magnetic resonance imaging-proton density fat fraction (MRI-PDFF) in non-diabetics with MAFLD at week 52, enrolled a total of 98 patients. Secondary endpoints included liver transaminases, fasting glucose, body mass index (BMI)/weight and waist circumference. The results of this study were published in 2024 by Cheung et al., who reported that patients receiving empagliflozin lost significantly more body weight and waist circumference and had lower fasting blood glucose than the control group receiving placebo drug [82,83].

### 2.5. Peroxisome Proliferator-Activated Receptor (PPAR) Agonists

A family of nuclear receptors, the peroxisome proliferator-activated receptors (PPARs), are involved as transcription factors in various processes of lipid and glucose metabolism and inflammation [84,85]. Moreover, they appear to be involved in fibrotic processes, as agonism of the PPAR isotope gamma (γ) leads to an inhibition of the pro-fibrotic effect of hepatic stellate cells in the liver, which are activated in the course of NASH progression [86]. Generally, in MAFLD, PPARs are dysregulated, and the effect of the agonist for PPAR α/γ receptor, pioglitazone, is associated with improvement in steatosis, inflammation and hepatic biomarkers, making it a very compelling choice for MAFLD therapy [84,87,88]. This drug belongs to the drug group of thiazolidinedione commonly called “glitazone” and is indicated for T2DM due to its efficacy in lowering blood glucose levels by enhancing insulin resistance [89]. Moreover, treatment with pioglitazone is already included in several guidelines as a possible treatment option for patients with T2DM and proven MASH [90]. On the other hand, it should be pointed out that the clinical use of pioglitazone is limited due to its potential side effects including weight gain, bladder cancer, fluid retention which can cause congestive heart failure in patients having cardiomyopathy and an enhanced risk of bone loss and distal bone fractures in postmenopausal women, and therefore careful selection of patients for pioglitazone therapy is required [87,91]. The assessment of the efficacy of oral pioglitazone on hepatic steatosis and liver function tests over 24 weeks and its safety was conducted in a placebo-controlled RCT (NCT01068444) in 90 Taiwanese participants with MASH. In this study, a significant drop in ALT, MAFLD activity score (MAS) and liver fat was observed, and pioglitazone was found to be generally safe. In addition, 46.7% of patients in the pioglitazone group showed an amelioration of MASH without aggravation of fibrosis [89,92]. A meta-analysis analyzing the available trials on pioglitazone confirmed that pioglitazone is an effective treatment for prediabetic or diabetic patients with MAFLD, as it reduces steatosis, liver function and inflammation. However, the effect on fibrosis stage is not yet clear, as no significant improvement in liver fibrosis was observed [93]. Currently, scientists at the University of Florida in the United States are also investigating the efficacy and safety of pioglitazone in the RCT known as AIM 2, but at a low dose (15 mg per day) and in patients with T2DM and MASH, with the plan of enrolling 166 patients and a duration until the end of 2027 (NCT04501406) [89].

Another member of the PPAR family being considered for the treatment of MAFLD is the dual PPAR-α and PPAR-γ agonist saroglitazar. In March 2020, following successful completion of trials in India, this drug was approved for MASH in India, but not yet in the other countries [89,94]. In the United States, saroglitazar has been granted marketing authorization for the treatment of dyslipidemia and hypertriglyceridemia in diabetic patients [95]. Furthermore, this drug showed confirmed beneficial effects on the liver in a placebo-controlled RCT (NCT03061721) named EVIDENCES IV, conducted in the United States on 106 patients with MAFLD or MASH. Liver fat content (measured with MRI-PDFF), ALT, adiponectin, triglyceride and insulin resistance were significantly reduced at a dose of 4 mg saroglitazar magnesium [96]. Saroglitazar magnesium is currently being investigated among patients with MASH and fibrosis in an ongoing placebo-controlled RCT (NCT05011305), which is expected to end next year with a total of 240 enrolled participants. The primary objective of this study is to investigate the resolution of MASH without worsening fibrosis after 52 weeks of saroglitazar therapy. One of the secondary objectives is to determine whether saroglitazar can improve liver fibrosis without worsening liver inflammation, steatosis or ballooning [86,97].

The fact that PPARs are currently a very appealing target in MAFLD is also confirmed by the new competitor lanifibranor. This drug is a pan-PPAR agonist that can bind to different regions of the PPAR α-, δ- and γ-ligand domain and thereby act as an agonist on all three PPAR isoforms [98,99]. Owing to this mechanism of action, lanifibranor may have a more potent effect on reducing inflammation, liver fibrosis and metabolic risk factors in MAFLD patients than a single PPAR agonist [100,101]. The NATIVE study is a phase 2b placebo-controlled RCT (NCT03008070) with a total enrollment of 247 participants, in which lanifibranor showed superiority over placebo in reducing the SAF-A score (activity part of the Steatosis Activity Fibrosis score) by at least two points without deterioration of fibrosis after 24 weeks of therapy [102,103]. Those favorable outcomes led to the launch of the NATIV3 study, a phase 3 placebo-controlled RCT (NCT04849728), which aims to enroll 1000 patients with active NASH and liver fibrosis (stage F2/F3) and to be completed by October 2026 (Figure 2). This study is designed to measure the resolution of MASH with improvement in fibrosis at week 72. The positive results of this study could bring lanifibranor a significant step closer to approval [104,105]. In Table 1 we have presented these two studies for lanifibranor together with the latest studies for other study drugs mentioned prior in this review.

### 2.6. Review Limitation

To reflect the accepted new terminology and to facilitate readability of this paper, the new term MASH has been used instead of NASH. This is also the main limitation of this review, as the RCTs mentioned included patients based on NASH criteria, which are not the same as those for MASH. The main difference is that NASH patients do not necessarily have at least one of the five cardiometabolic risk factors (obesity, T2DM, hypertension, elevated plasma triglycerides and decreased plasma HDL) [106]. It is therefore uncertain whether the study results would be significantly different if patients with MASH criteria were included in the study. On the other hand, it is important to emphasize that the term steatohepatitis as well as the criteria for fibrosis stages were preserved in both terminologies. In addition, patients with steatosis without cardiometabolic risk factors or other possible causes are considered cryptogenic and candidates for possible MAFLD who would benefit from regular reassessment [8,106,107]. The sponsor of the MAESTRO-NASH study (NCT03900429) for resmetirom, which involved patients with NASH and fibrosis, announced along with the study results that the new terminology for NASH is MASH [20]. However, to correctly adopt the MASH terminology, studies would need to enroll patients with MASH, which is expected in future RCTs for this indication.

## 3. Future Directions

In the future, grouping MAFLD patients according to their liver histology (steatosis, steatohepatitis and fibrosis) may be less useful than with respect to main pathological mechanism involved, which is more suitable for predicting disease outcome [8]. A similar conclusion was reached in a large prospective cohort study in China, which showed that the higher mortality of MAFLD patients depended on the presence of various metabolic risk factors such as type 2 diabetes mellitus, obesity and other comorbidities. Based on this study result, the authors concluded that in the future it may be necessary to sub-categorize MAFLD depending on the severity of a patient’s metabolic syndrome symptoms in order to ensure more effective treatment [108]. In addition, given that MAFLD is a multisystemic disease, a multidisciplinary approach is required, and it is likely that monotherapy (in combination with lifestyle modification) to treat MAFLD will not provide complete treatment success. To achieve this, combination therapy targeting different pathological pathways and organ systems needs to be more intensively explored [17,109]. Nevertheless, there is still a relatively long way to go before such combination therapies can be introduced into clinical practice, as there are several points to consider in addition to extensive regulatory requirements: Firstly, the use of monotherapy in patients must be well established; secondly, clinical trials must be very well designed and carried out on a large number of participants; and thirdly the dose and safety of such preparations must be examined extremely carefully [110]. Last but not least, the treatment of patients who do not respond to “conventional” treatment or are not compliant must also be considered, so that it would be useful to elaborate a more personalized treatment approach [111].

## 4. Conclusions

MAFLD is a potentially life-threatening liver disease with a substantial impact on global health. Lifestyle intervention remains crucial, but as a single approach is generally not sufficiently effective. Hence, there is an immense need for approved treatment options to enhance patient outcomes. Researchers around the globe, especially in the United States, are making great efforts to develop new molecules for MAFLD or to take a “shortcut” in drug development and regulatory approval processes and expand the indication of drugs already approved for another medical condition. The most trialed agents appear to be hypoglycemics, with study results expected in the next few years. However, the main drivers towards marketing authorization are the agents which can significantly ameliorate liver fibrosis, which is a critical factor in the progression of MAFLD. Some drugs, such as pioglitazone, have been shown to be effective in lowering MAFLD parameters, but the evidence of their effect on fibrosis is still lacking. All in all, the race among pharmaceutical companies seeking marketing authorization for MAFLD is becoming increasingly intense; however, the need for approved pharmacotherapies and treatment approaches is yet to be fulfilled.

## Figures and Tables

**Table 1:** Completed and ongoing RCTs for metabolic-associated fatty liver disease (MAFLD) treatment. ^1^ The primary endpoint was already achieved. ^2^ Detailed study results are pending. RCT = randomized clinical trial, N/A = not applicable as no published results yet, SAF-A (activity part of the Steatosis Activity Fibrosis score), TGs = triglycerides, LDL = low-density lipoprotein, ALT = alanine aminotransferase. Adapted and actualized from Sangro et al. [69].

Study Drug	RCT	Sponsor	Country	Study End	Results
RESMETIROM (MGL-3196)	NCT02912260 (OLE)	Madrigal Pharmaceuticals, Inc. (West Conshohocken, PA, USA)	United States	2018	Reduction in fibrosis, LDL and TG levels.
	NCT03900429 (MAESTRO-NASH)			Ongoing	N/A
VK2809	NCT02927184	Viking Therapeutics, Inc. (San Diego, CA, USA)	United States	2019	Reduction in LDL-C, other hepatic lipid content and ALT.
	NCT04173065 (VOYAGE)			Ongoing	Reduction of hepatic fat content ^1^.
PEGOZAFERMIN (BIO89-100)	NCT04929483 (ENLIVEN)	89bio, Inc. (San Francisco, CA, USA)	United States	Ongoing	Reduction of fibrosis.
	NCT06318169 (ENLIGHTEN- Fibrosis)			Ongoing	N/A
EFRUXIFERMIN	NCT04767529 (HARMONY)	Akero Therapeutics, Inc. (South San Francisco, CA, USA)	United States	2022	Improvement of liver fibrosis by at least one stage, without MASH worsening.
	NCT06215716 (SYNCHRONY Histology)			Ongoing	N/A
	NCT06161571 (SYNCHRONY Real-World)			Ongoing	N/A
DAPAGLIFLOZIN	NCT05308160	National Taiwan University Hospital (Taipei City, Taiwan)	Taiwan	Ongoing	N/A
	NCT03723252 (DEAN)	Nanfang Hospital, Southern Medical University (Guangzhou, China)	China	2024	N/A
	NCT05459701	Rehab Werida, Damanhour University (Damanhour City, Egypt)	Egypt	Ongoing	N/A
EMPAGLIFLOZIN	NCT04642261	The University of Hong Kong (Pokfulam, Hong Kong, China)	China	2023	Reduction of body weight, waist circumference and fasting blood glucose.
SEMAGLUTIDE	NCT02970942	Novo Nordisk A/S (Plainsboro, NJ, USA)	United States	2020	Reduction of ALT level, liver inflammation and steatosis.
	NCT03357380		Germany	2020	
	NCT04822181 (ESSENCE)		United States	Ongoing	N/A
EFINOPEGDUTIDE (MK-6024)	NCT04944992 (MK-6024-001)	Merck Sharp & Dohme LLC (Rahway, NJ, USA)	United States	2022	Reduction in hepatic fat.
	NCT05877547 (MK-6024-013)			Ongoing	N/A
TIRZEPATIDE	NCT04166773 (SYNERGY-NASH)	Eli Lilly and Company (Indianapolis, IN, USA)	United States	2024	Resolution of MASH and no worsening of fibrosis. ^2^
SAROGLITAZAR	NCT03061721 (EVIDENCES IV)	Zydus Therapeutics Inc. (Pennington, NJ, USA)	United States	2020	Reduction of liver fat content, ALT, triglycerides, adiponectin and insulin resistance.
	NCT05011305			Ongoing	N/A
PIOGLITAZONE	NCT01068444	Kaohsiung Medical University Chung-Ho Memorial Hospital (Kaohsiung City, Taiwan)	Taiwan	2020	Reduction of liver fat content and inflammation. MASH resolution without fibrosis worsening.
	NCT04501406	University of Florida (Gainesville, FL, USA)	United States	Ongoing	N/A
LANIFIBRANOR (IVA 337)	NCT03008070 (NATIVE)	Inventiva Pharma (Daix, France)	United States	2020	Reduction of the SAF-A score by at least two points without fibrosis worsening.
	NCT04849728 (NATIV3)			Ongoing	N/A
